# 3D‐e‐Chem: Structural Cheminformatics Workflows for Computer‐Aided Drug Discovery

**DOI:** 10.1002/cmdc.201700754

**Published:** 2018-02-14

**Authors:** Albert J. Kooistra, Márton Vass, Ross McGuire, Rob Leurs, Iwan J. P. de Esch, Gert Vriend, Stefan Verhoeven, Chris de Graaf

**Affiliations:** ^1^ Centre for Molecular and Biomolecular Informatics (CMBI) Radboud University Medical Center (RadboudUMC) Nijmegen The Netherlands; ^2^ Division of Medicinal Chemistry, Faculty of Science, Amsterdam Institute for Molecules, Medicines and Systems (AIMMS) Vrije Universiteit Amsterdam Amsterdam The Netherlands; ^3^ BioAxis Research, Pivot Park Oss The Netherlands; ^4^ Netherlands eScience Center Amsterdam The Netherlands

**Keywords:** cheminformatics workflows, KNIME, ligand design, ligand repurposing, target prediction

## Abstract

eScience technologies are needed to process the information available in many heterogeneous types of protein–ligand interaction data and to capture these data into models that enable the design of efficacious and safe medicines. Here we present scientific KNIME tools and workflows that enable the integration of chemical, pharmacological, and structural information for: i) structure‐based bioactivity data mapping, ii) structure‐based identification of scaffold replacement strategies for ligand design, iii) ligand‐based target prediction, iv) protein sequence‐based binding site identification and ligand repurposing, and v) structure‐based pharmacophore comparison for ligand repurposing across protein families. The modular setup of the workflows and the use of well‐established standards allows the re‐use of these protocols and facilitates the design of customized computer‐aided drug discovery workflows.

## Introduction

There is a need for eScience technologies to process the large volumes of rapidly generated, heterogeneous^1^ protein–ligand interaction data into computational models that enable the design of efficacious and safe medicines.^2^ The ChEMBL database (version 23), for example, contains over 14 million data entries on 11 500 protein targets, of which 4600 human, covering 1.7 million unique compounds.^3^ The Protein Data Bank (PDB, accessed October 21, 2017) contains more than 130 000 structures with nearly 24 000 small molecules covering 67 000 unique protein–ligand complexes.^4^ Currently 20 000 human proteins have been deposited in Swiss‐Prot^5^ (version 2017_10), of which 3300 proteins are also present in ChEMBL. Comparison of the protein, ligand, and bioactivity data in ChEMBL, PDB, and UniProt indicates that structural information is lacking for more than 95 % of the protein–ligand pairs for which bioactivity data has been reported, and for more than 75 % of the human proteins for which sequence information is available. In silico chemogenomics^6^ and computer‐aided drug discovery methods can be used to predict protein–ligand interactions in order to fill these bioactivity‐structure and sequence‐structure gaps, identify new protein–ligand pairs, and design new ligands.6b, 7 The success rate of such methods strongly depends on the efficient integration of chemical, pharmacological and structural data to train, optimize, and evaluate ligand‐ and protein‐based models.6b, 7a,7b An effective approach to accomplish this is through the development of scientific workflows^8^ that facilitate the standardization of protocols,7c the integration of data and analyses, and re‐use of parts of protocols to customize, extend, or design new workflows for different targets or applications.^9^ KNIME^10^ and Pipeline Pilot^11^ are established workflow managers in the field of cheminformatics and computer‐aided drug discovery, with a growing number of users.^8^ Several ligand‐based workflows have been reported that combine chemical and biological data sources for ligand‐based target prediction.^12^ Few structure‐based workflows have been reported, including protocols for pharmacophore screening,^13^ structure‐based ligand optimization,^14^ as well as combined ligand‐ and protein‐based ligand repurposing.^15^ Several of the tools in the reported workflows, however, use commercial computer‐aided drug discovery software that is not accessible without a paid license.15b, 16 Most freely available cheminformatics tools^17^ (nodes) that can be run within these workflows focus on small molecules^18^ and the number of nodes that use freely available structure‐based approaches is relatively scarce.

The current work describes the integration and analysis of several chemical, biological, and structural data types in workflows that can be used for: i) structure‐based bioactivity data mapping, ii) structure‐based identification of scaffold replacement strategies for ligand design, iii) ligand‐based target prediction, iv) protein sequence‐based binding site identification and ligand repurposing within a protein family, and v) structure‐based pharmacophore comparison for ligand repurposing across protein families.

The flexible workflows and protocols presented here can be used as templates for the standardization of protocols, the integration of data and analyses, and can readily be reused or extended for the creation of new computer‐aided drug discovery workflows for other protein targets and applications. The cases will focus on two of the pharmaceutically most relevant protein targets, namely G protein‐coupled receptors (GPCRs) and kinases.

**Figure 1:**
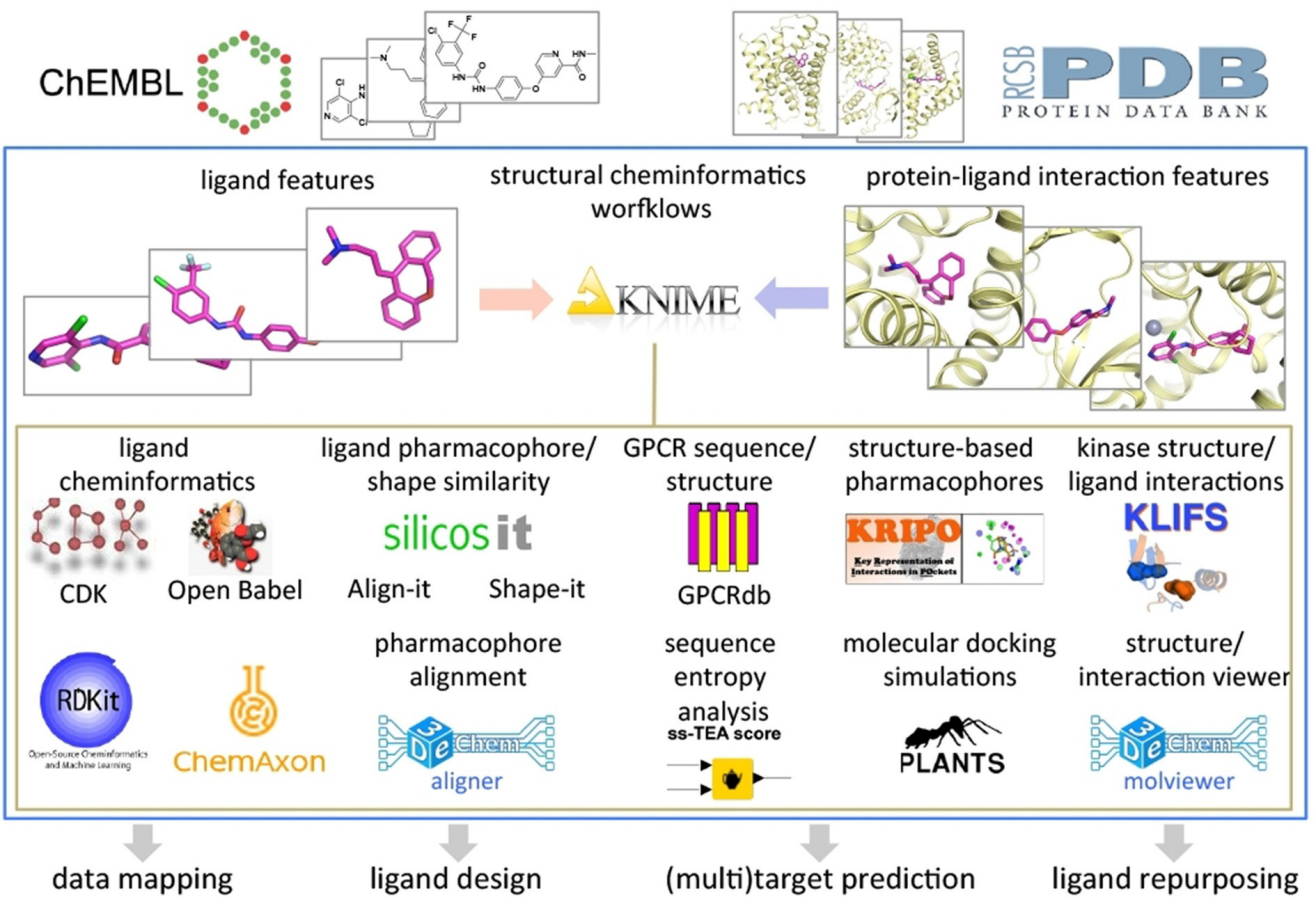
Overview of structural cheminformatics tools and workflows for computer‐aided drug discovery applications described in the current study. Pharmacological (ChEMBL)^3^ and structural (PDB)^4^ data on protein–ligand interactions are integrated and complemented by structural chemogenomics analyses of ligand, protein, and protein–ligand interaction features by the combination of different KNIME nodes, including small molecule ligand cheminformatics toolkits (e.g., CDK,^17^, ^19^ ChemAxon,^20^ Openbabel,^21^ RDKit),^22^ web service nodes to extract information from GPCR (GPCRdb)^23^ and kinase (KLIFS)^24^ focused databases, and nodes to perform ligand shape‐based (Shape‐it),^25^ ligand pharmacophore‐based (Align‐it),^25^ and protein pharmacophore‐based (KRIPO)^26^ similarity searches, molecular docking simulations (PLANTS),^27^ amino acid sequence entropy analyses (ss‐TEA),^28^ pharmacophore alignments (aligner), and to visualize protein–ligand complexes and pharmacophores (molviewer). Workflows for structure‐based bioactivity data mapping, ligand design, target prediction and ligand repurposing are described in the current work and provided as Supporting Information.

Moreover, this work presents new KNIME nodes that enable the analysis and prediction of protein–ligand interactions using freely accessible structural cheminformatics tools, including: i) web service nodes to extract and combine data from GPCR (GPCRdb)^23^, ^29^ and kinase^30^ (KLIFS)^24^ focused databases, ii) nodes to set up, run, and analyze results of structural pharmacophore‐based protein binding site comparison (KRIPO),^26^, ^31^ ligand shape‐based (Shape‐it)^25^ and pharmacophore‐based (Align‐it)^25^ comparison, and molecular docking simulation (PLANTS)^27^ tools, and, iii) new KNIME nodes to perform amino acid sequence entropy analyses (ss‐TEA),^28^ align (aligner), read, and write pharmacophores (pharmacophores), and visualize protein–ligand complexes and pharmacophores in 3D (molviewer) (Figure 1).

All nodes and tools used to perform the analyses described in the current work are available as community contributions in KNIME under “3D‐e‐Chem” (https://www.knime.com/3d-e-chem-nodes-for-knime), the source code for all nodes and all workflows themselves are available via GitHub (https://github.com/3D-e-Chem/workflows), and everything is also embedded within an updated version of our 3D‐e‐Chem virtual machine^31^ (https://3d-e-chem.github.io/3D-e-Chem-VM/). This enables all users to download, apply, customize, and extend the workflows to their own protein targets of interest in order to answer different chemogenomics or drug discovery related questions.

## Results and Discussion

### Structure‐based bioactivity data mapping of kinase inhibitors

**Figure 2:**
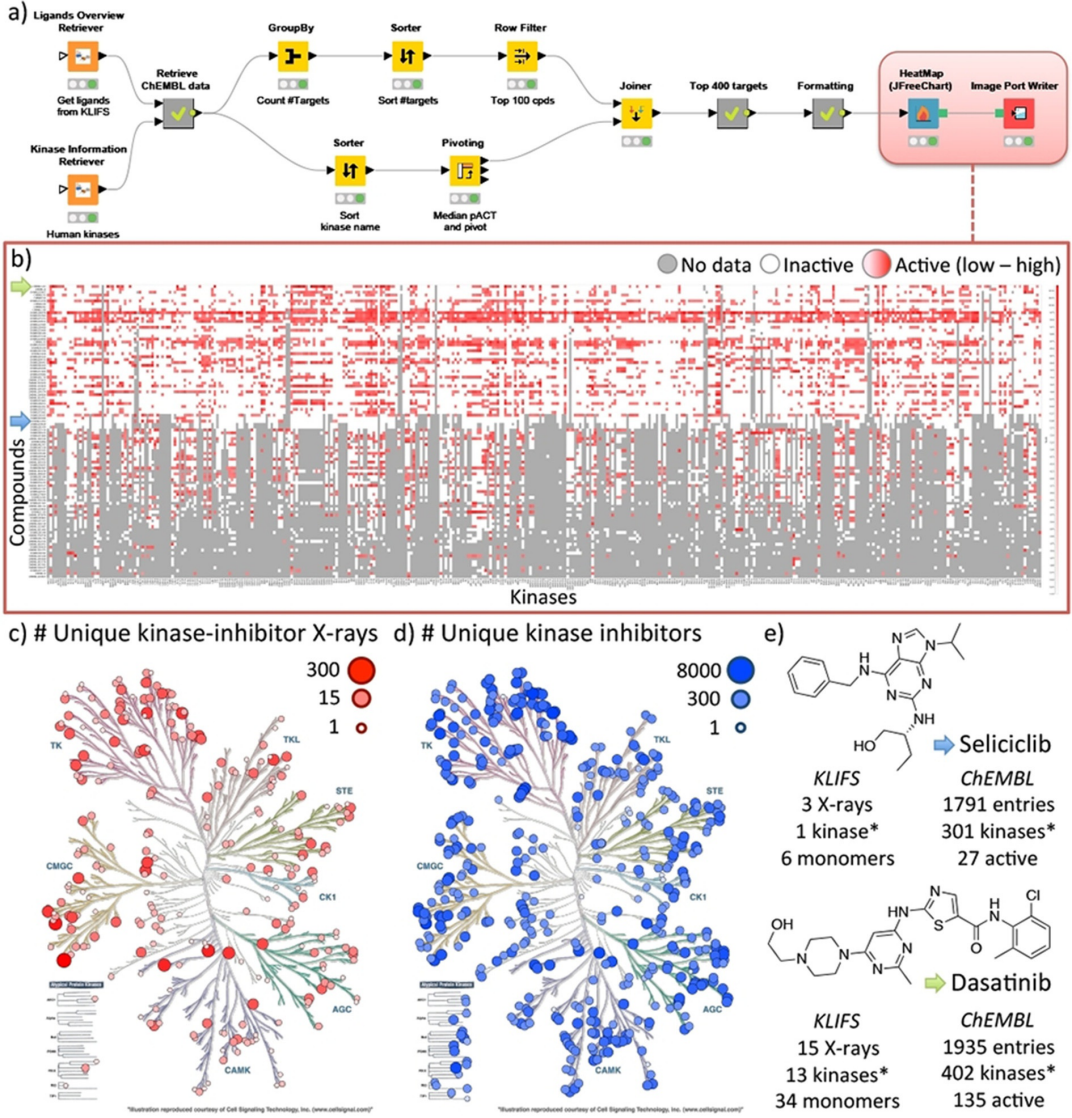
Structure‐based bioactivity data mapping workflow (A) of kinase inhibitors using both the KLIFS and the ChEMBL database. The heatmap (B) shows the bioactivity profile for the top 100 co‐crystallized kinase ligands with the largest amount of data available for the top 400 kinases. The kinomes, created with KinMap,^32^ show the number of unique kinase‐inhibitor complexes based on KLIFS (C) and the number of unique kinase inhibitors based on ChEMBL (D). The data accumulated in this workflow are summarized (E) for two well‐known kinase inhibitors, namely Seliciclib and Dasatinib (indicated with a blue and green arrow, respectively on the *Y*‐axis of the heatmap). *Only human kinases are listed.

Protein–ligand crystal structures provide information regarding protein–ligand interactions and protein conformations, whereas bioactivity data provides insight into the binding affinity or functional effect. The integration of structural and bioactivity data allows one to interpret differences and similarities in bioactivity (e.g., affinity cliffs) to ligand binding modes, specific protein–ligand interactions, and to extrapolate these insights to other protein targets. In the next workflow (Figure 2) we have combined bioactivity data from ChEMBL and (structural) kinase data from KLIFS to create a matrix of available bioactivity data on human kinases for all co‐crystallized kinase ligands.

**Protocol**:

1. Collect protein information and the molecular structures of co‐crystallized ligands (here from KLIFS)
2. Retrieve the available bioactivity data for the ligands (here from ChEMBL)
3. Clean, curate, and process the bioactivity data
4. Selection of the compounds and kinase targets of interest
5. Formatting and visualizing the data

The molecular structures of all 2552 unique co‐crystallized small molecule kinase inhibitors were collected via KLIFS nodes (KLIFS accessed August 18^th^, 2017) in SMILES format. The InChIKeys of the inhibitors were subsequently used to retrieve the ChEMBL IDs for the compounds (1583 matches) including all corresponding bioactivity data (166 976 data points). Using the human kinase list from KLIFS all bioactivity data was reduced to solely the human kinome (86 601 data points for 432 kinases). The top 100 compounds with the largest number of available bioactivity data (excluding single concentration measurements) for kinases^30^ was then selected together with the top 400 kinases and the median log value of the bioactivity data for each unique compound–kinase pair. The data was then transformed into a matrix and visualized as a heatmap using the JFreeChart HeatMap node. The heat map shows clear differences in the bioactivity profiles between kinase inhibitors and highlights promiscuous and selective compounds as well as the gaps in the bioactivity matrix. This workflow illustrates a simple, yet powerful, method of complementing a structure‐based view of kinase inhibitors with the available pharmacological data for more advanced structural chemogenomics applications (Figure 2).

### Scaffold replacements for kinase ligand design

**Figure 3:**
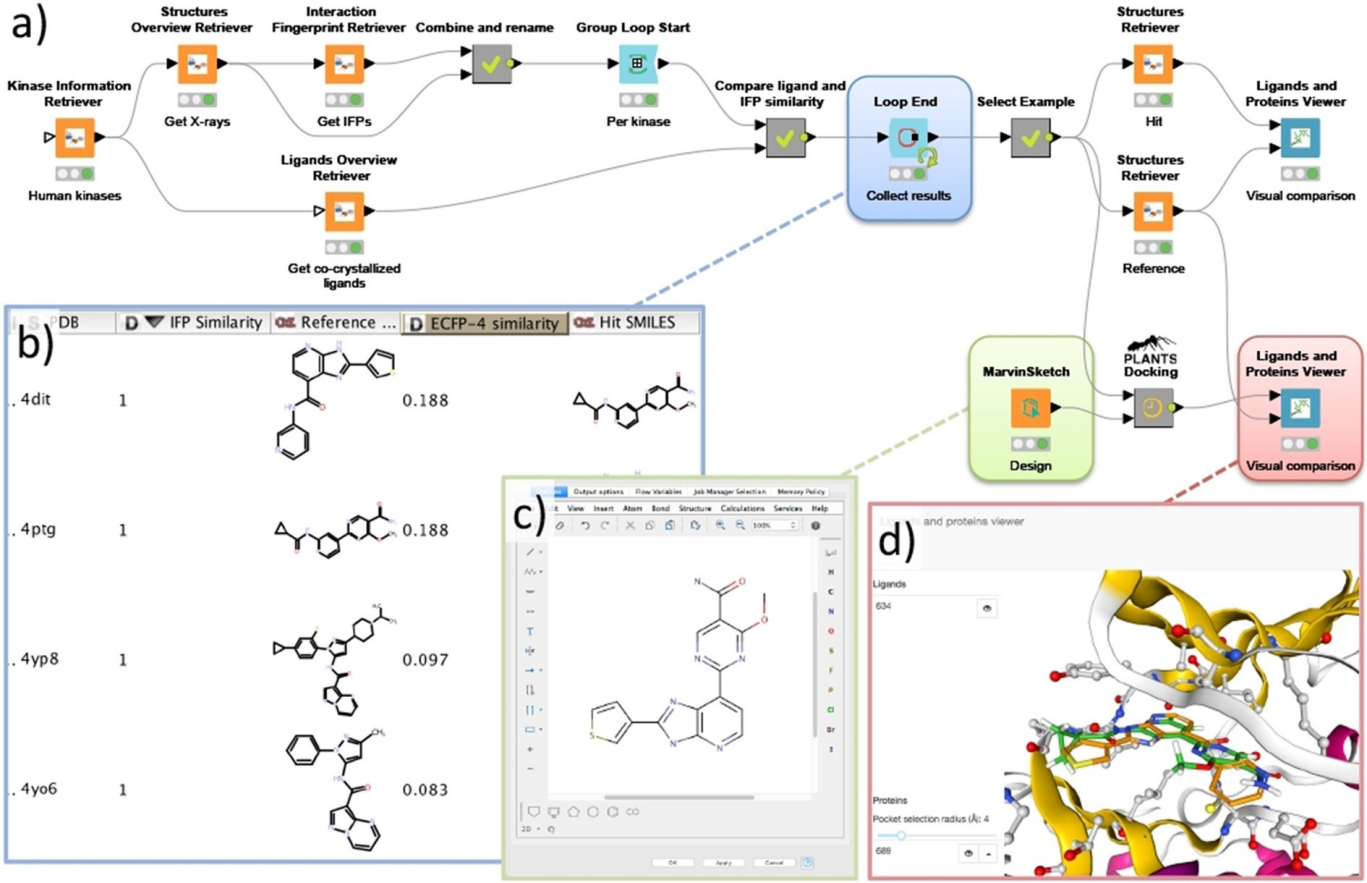
A workflow (A) for the identification of potential scaffold replacements for kinase inhibitors while maintaining the protein–ligand interaction profile by combining protein–ligand interaction fingerprint (IFP) similarity with ligand‐based dissimilarity (ECFP‐4) analyses. The scaffold hop between an imidazopyridine inhibitor (PDB ID: http://www.rcsb.org/pdb/explore/explore.do?structureId=4DIT)^35^ and a carboxamide inhibitor (PDB ID: http://www.rcsb.org/pdb/explore/explore.do?structureId=4PTG),^36^ shown as the first entry in the table overview (B), was used to design a merged molecule (C). This design was docked into GSK3B (PDB ID: http://www.rcsb.org/pdb/explore/explore.do?structureId=4PTG)^36^ using the PLANTS nodes and visualized in the Ligand and Protein Viewer (D).

Scaffold hopping is a common approach in which a part of a known active compound is changed while trying to maintain the binding affinity and binding mode of the original compound in order to obtain better ADMET/PKPD or physicochemical properties or to escape patent infringement.^33^ In the next workflow (Figure 3) protein–ligand interaction similarity6a, 34 as well as chemical similarity is used to identify molecular pairs with a low chemical similarity but a high interaction similarity, thereby providing interesting starting points for the design of hybrid molecules that have a high probability of maintaining their binding mode.

**Protocol**:

- Collect structural information, protein–ligand interaction fingerprints (IFP), and molecular structures of the co‐crystallized ligands (here from KLIFS)
- Perform full pairwise ligand‐based similarity and IFP similarity analyses
- Filter the data by selecting ligand pairs with a low molecular similarity and a high interaction similarity
- Obtain the aligned structures and compare the binding modes of the molecule pairs of interest
- Design a scaffold hop based on the selected molecule pair and dock them into the desired protein kinase
- Visually evaluate the obtained binding modes, compare their interaction fingerprints, or perform another binding mode comparison technique.

Starting from the KLIFS nodes all structural information on human kinases (7552 unique monomers) was downloaded including the kinase‐inhibitor interaction fingerprints (IFP) and the SMILES of the co‐crystallized kinase inhibitors. Subsequently, a group loop is started that processes all structures per individual kinase. Within the loop, a pairwise interaction‐based IFP6a, 34 and ligand‐based ECFP‐4^37^ comparison is performed for all complexes of each kinase. The combinations are subsequently filtered for ligand pairs with a low chemical similarity (ECFP‐4 Tanimoto score <0.26) and a high interaction similarity (IFP Tanimoto score >0.75), that is, all chemically distinct ligand pairs that do have similar interactions with the kinase target are selected. From the resulting list of pairs, an imidazopyridine inhibitor (PDB ID: http://www.rcsb.org/pdb/explore/explore.do?structureId=4DIT)^35^ and a carboxamide inhibitor (PDB ID: http://www.rcsb.org/pdb/explore/explore.do?structureId=4PTG)^36^ in complex with GSK3B with a very low ligand similarity (Tanimoto ECFP‐4=0.188) and an identical protein–ligand interaction pattern (Tanimoto IFP=1.0) were selected as an example for further inspection. From both structures, the KLIFS aligned full monomer and ligand were download and subsequently visualized using the Ligands and Proteins Viewer showing the overlay of the ligands in the GSK3B binding site. These two kinase inhibitors were subsequently used to design a hybrid compound drawn in the MarvinSketch node. Finally, this design was docked into the GSK3B binding site (PDB ID: http://www.rcsb.org/pdb/explore/explore.do?structureId=4PTG) using the newly developed PLANTS^27^ docking nodes. Upon visual inspection of the obtained binding modes within the Ligands and Proteins viewer, a highly conserved binding mode of both parts of the hybrid design is observed. Within this workflow the chemical dissimilarity is complemented with protein–ligand interaction patterns to identify distinct molecules with similar mechanisms of binding. This combination of techniques provides new opportunities for molecular design based on known ligands and the workflow could, for example, be rewired and extended for more advanced fragment‐based replacement approaches.

### Ligand‐based cross‐reactivity prediction

**Figure 4:**
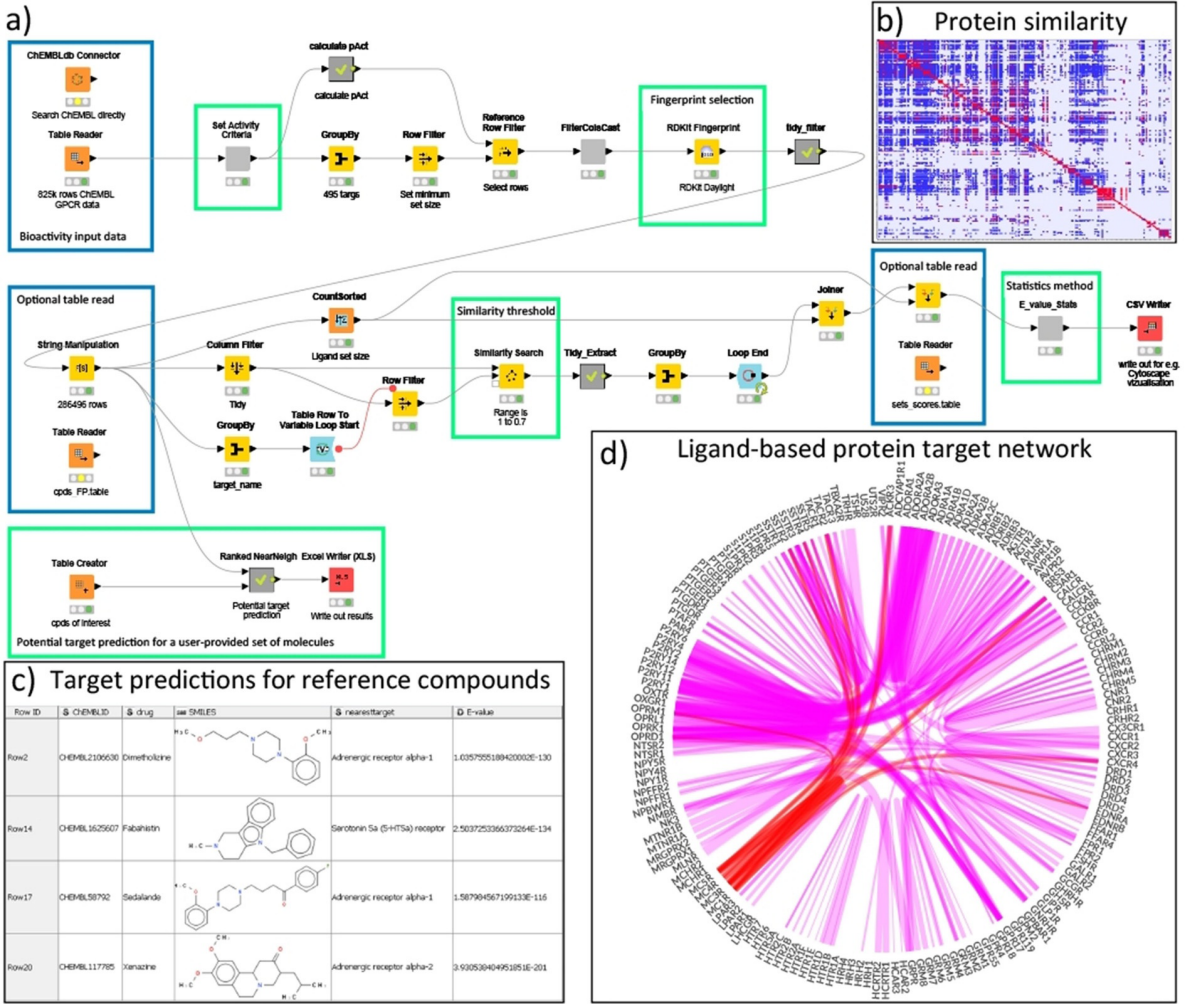
Ligand‐based GPCR cross‐reactivity workflow (A) with selected output (C) from the nearest neighbor calculation of four of the five reference compounds. Blue boxes highlight areas where recalculated tables are provided and may be used for faster and more efficient processing. Green boxes show areas for user input and adjustment. The heatmap (B) summarizes the ligand‐based similarity overlap for all provided GPCR ligands. The protein target network (D) highlighted in a flareplot^38^ shows the top 500 associations between protein targets based on their shared ligand similarities (line thickness indicates the significance), the associations of the melanocortin receptors are highlighted in red.

The derivation of similarity measures between different protein receptors may be used to explore cross‐reactivities and to explore the potential for compounds to display (useful) polypharmacology. The PP_GPCR (protein–protein association GPCR) workflow (Figure 4 A) follows methodologies used in previous efforts^39^ to explore the relationships between protein targets using ligand topology. This chemocentric approach involves describing the sets of ligands for each protein target by chemical fingerprint descriptors,^40^ and comparing the sets with each other to derive similarities between protein targets. With this approach, one can derive protein–ligand and protein–protein associations ranging from biologically expected to less obvious.

**Protocol**:

- Collect available bioactivity data for a protein family or (full) set of proteins of interest
- Clean, curate, process, and filter the bioactivity data
- Calculate ligand‐based fingerprint descriptors for each compound
- *Goal 1: Protein–protein association prediction*
- ○ Perform an all‐against‐all comparison of the fingerprints and select relevant hits based on a user‐definable cutoff
- ○ Group the number of hits per protein target pair and calculate an E‐value
- ○ Output of the results for visualization in, for example, Cytoscape^41^ or flareplots.^38^
- *Goal 2: Identification of potential protein targets for small molecules*
- ○ User input of the small molecules of interest and calculate their ligand‐based fingerprint descriptors
- ○ Perform a fingerprint comparison against the protein dataset and select hits based on a user‐definable cutoff
- ○ Group the number of hits per protein target and calculate an E‐value

The protocol is applicable to any combination of data sets with unknown distributions of structures and biological activity values, user intervention to vary thresholds, similarity measures, fingerprints and statistical approaches is made possible. The PP_GPCR workflow reads in data from a public data source, ChEMBL, for all non‐olfactory GPCR receptors as derived from the GPCRdb.^23^ Various filters for allowed activity type (EC_50_, IC_50_, AC_50_, *K*_b_, *K*_D_, *K*_i_) and threshold activity (pAct ≥5) are applied, a minimum compound set size of 5 is required, and a restriction on the number of calculated rotatable bonds (maximum of 18) is used to limit the number of very large, flexible compounds. The latter is performed as in our experience the presence of large numbers of peptide/peptoid compounds can lead to some targets being routinely overrepresented in later comparisons. Fingerprint descriptors (in this case RDKit: Daylight‐like topological fingerprint) were calculated for each compound and the similarities between the receptor sets were determined using a user‐definable threshold for similarity, here set to a minimum of 0.7. Use of the raw similarities and set size following Keiser39a allowed the calculation of E‐values, used to rank the similarity between protein targets. The similarities between receptors are viewable as a KNIME Table and Excel File. To highlight some of the identified similarities the top 500 protein associations were visualized in a flareplot^38^ (Figure 4 D) and a heatmap (Figure 4 B). The melanocortin receptors, for example, show links with opioid, endothelin, chemokine and somatostatin receptors. These associations have previously been explored by Quillan et al.^42^

The PP_GPCR workflow may also be used to calculate potential targets/cross‐activities for individual compounds. A compound may be entered into the workflow or, if already present in the data, simply extracted and compared with the fingerprints already present allowing the calculation of the statistical significance and ranking by E‐values. To analyze the predictive ability of the PP_GPCR workflow, the workflow was applied to five reference structures taken from Keiser et al.39b with an experimentally validated GPCR affinity (*K*_i_<1000 nm). Using the default similarity cut‐off of 0.7, for four of the five compounds (Sedalande, Dimetholazine, Xenazine and Fabhistine) previously predicted activities were reflected in the top‐five nearest neighbors in the PP_GPCR workflow (Figure 4 C). Lowering the similarity cut‐off increases the likelihood of detecting further nearest neighbors at the expense of a larger number of hits.

### Sequence‐based ligand repurposing within a protein family

**Figure 5:**
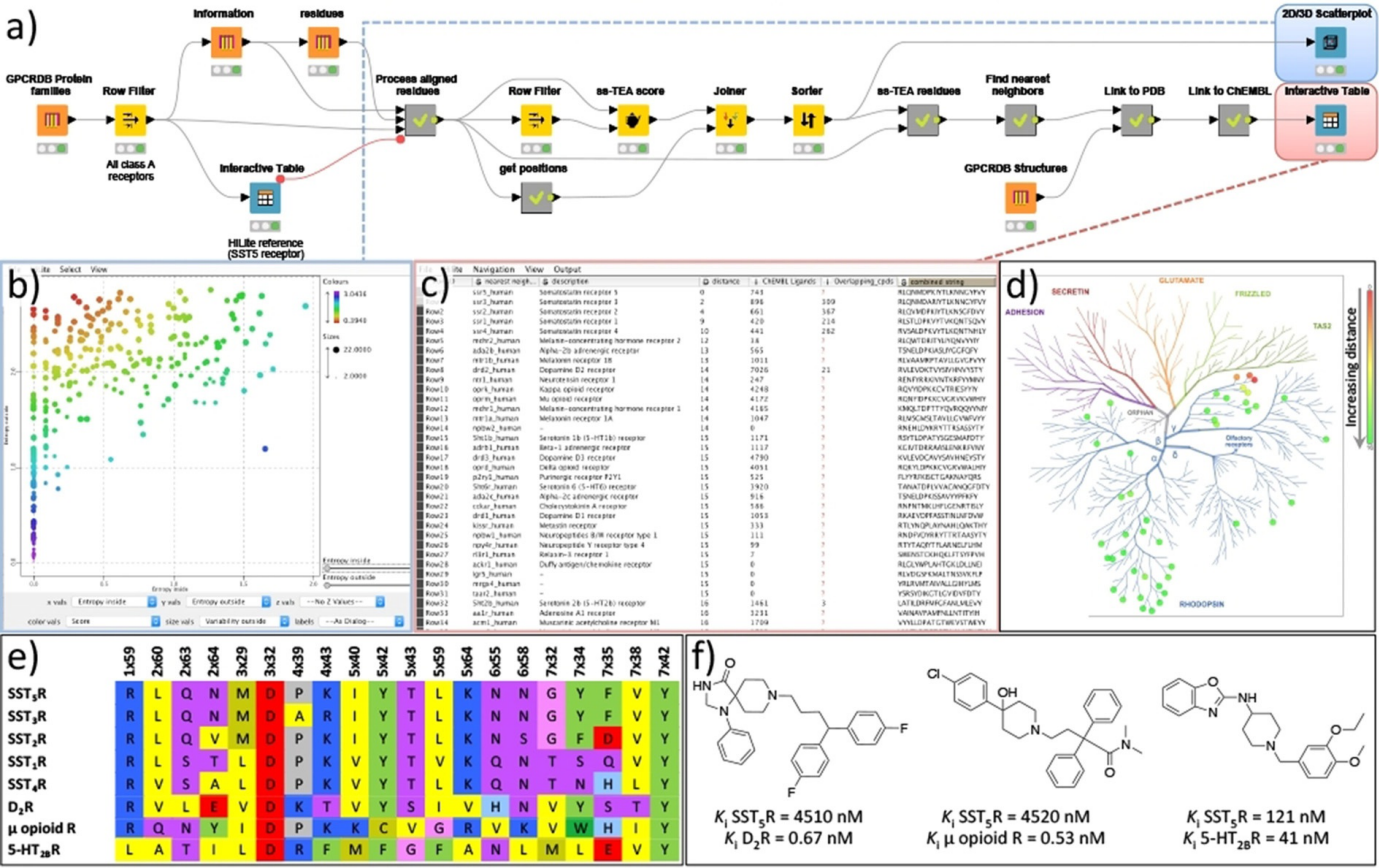
Workflow (A) for the identification of ligand repurposing possibilities using a sequence‐based double entropy analysis (ss‐TEA). This example shows the identification of the opioid, serotonin, and dopamine receptors as potential repurposing targets for somatostatin type 5 inhibitors, which was retrospectively verified using ChEMBL data (C) and a literature search (F). The scatterplot (B) shows the internal entropy (X‐axis) versus the external entropy (Y‐axis) for each residue and is colored by the ss‐TEA score (the lower the more significant). Part of the summarized analysis results are shown in (C) the interactive table viewer and (D) the identified nearest proteins for SST_5_R are shown in the phylogenetic tree of human GPCRs. (E) A sequence alignment of solely the residues (using the Ballesteros–Weinstein residue numbering scheme)^45^ identified with ss‐TEA for the somatostatin receptors and highlighted cross‐reactivity targets.

Sequence‐based identification of key residues for a specific protein can help with the identification of binding site residues or residues that are linked to a specific receptor function. More importantly, this information can be exploited for ligand repurposing as proteins that share similarity for these key residues can potentially bind similar ligands.^43^ In this workflow (Figure 5) we use a double entropy sequence analysis method (ss‐TEA) to identify these key residues, and perform a sequence‐based comparison for these residues to identify similar proteins (within the same protein family) as potential candidates for ligand repurposing.^44^

**Protocol**:

- Create or obtain a large sequence alignment for a protein family
- Selection of the protein subfamily of interest
- Perform the double entropy ss‐TEA analysis for identification of key residues for the selected subfamily
- Extract the aligned key residues and perform a sequence comparison to identify nearest neighbors
- Collect additional ligand and bioactivity data for the nearest neighbors

The workflow begins by gathering a complete list of all class A GPCR families (300), all class A GPCRs (11 731), and the aligned and numbered protein residues for each GPCR (4 536 590 in total) using the GPCRdb^23^ nodes. The structure‐based residue numbering was then used to obtain a matrix with the position‐based alignment of all GPCR residues. At this point, the user can inspect the table of GPCR families and highlight the GPCR receptor/subfamily of interest using an interactive table viewer. The user selection, in this case the Somatostatin receptor type 5 (SST_5_R), is then used to create a subfamily (i.e., reference group) as input for the double entropy analysis by the ss‐TEA node. All residue positions are scored according to the entropy within the subfamily (internal entropy) compared to the entropy outside the subfamily (external entropy). The 20 residue positions within the seven transmembrane helices with the lowest score (the residues with a low internal entropy, but a high external entropy) were selected for further processing. These residues have a high conservation of a residue within a subfamily but a low conservation outside a subfamily, which is an indication of the subfamily‐specific relevance of the residue for, for example, ligand recognition or receptor function. For visualization of the results, a scatterplot is created displaying the internal versus the external entropy with all residue positions (each dot) colored according to their ss‐TEA score (Figure 4 C). Subsequently, an alignment of solely the selected 20 residues is generated and used to calculate the sequence identity of the human GPCR of the subfamily to all human GPCRs. The nearest 50 GPCRs based on this ss‐TEA sequence alignment are selected and shown in an interactive table viewer as potential candidates for ligand repurposing and complemented by a list of available crystal structures in the PDB. Moreover, all ChEMBL bioactivities for each receptor are obtained and the number of active inhibitors annotated in ChEMBL is listed, including the number of known ligands that have both an affinity for the identified receptor as well as for the reference receptor. For the SST_5_R this selection of GPCRs logically contains the other somatostatin receptors and the closely related opioid receptors, but also the more distant dopamine as well as serotonin receptors (Figure 4). This matches with the known cross‐reactivity of some SST_5_R inhibitors for the μ opioid receptor, as well as the dopamine D_2_ receptor (D_2_R) and the serotonin 2B receptor (5‐HT_2B_R), which are also identified by the cross‐reactivity assessment using the ChEMBL bioactivities of the known SST_5_R inhibitors (see Figure 4 C,D). This is, for example, demonstrated by the cross‐reactivity of the marketed drugs Fluspirilene (a D_2_R antagonist) and Loperamide (a μ opioid agonist) on SST_5_R. Vice versa, a series of benzoxazole SST_5_R inhibitors showed nanomolar affinities for 5‐HT_2B_R (Figure 4 F). All these receptors share the key ionic anchor D^3.32^ (Figure 4 E) within the selected residues, which was deemed essential for the ligand recognition.^46^

### Structure‐based pharmacophore comparison for ligand repurposing across protein families

**Figure 6:**
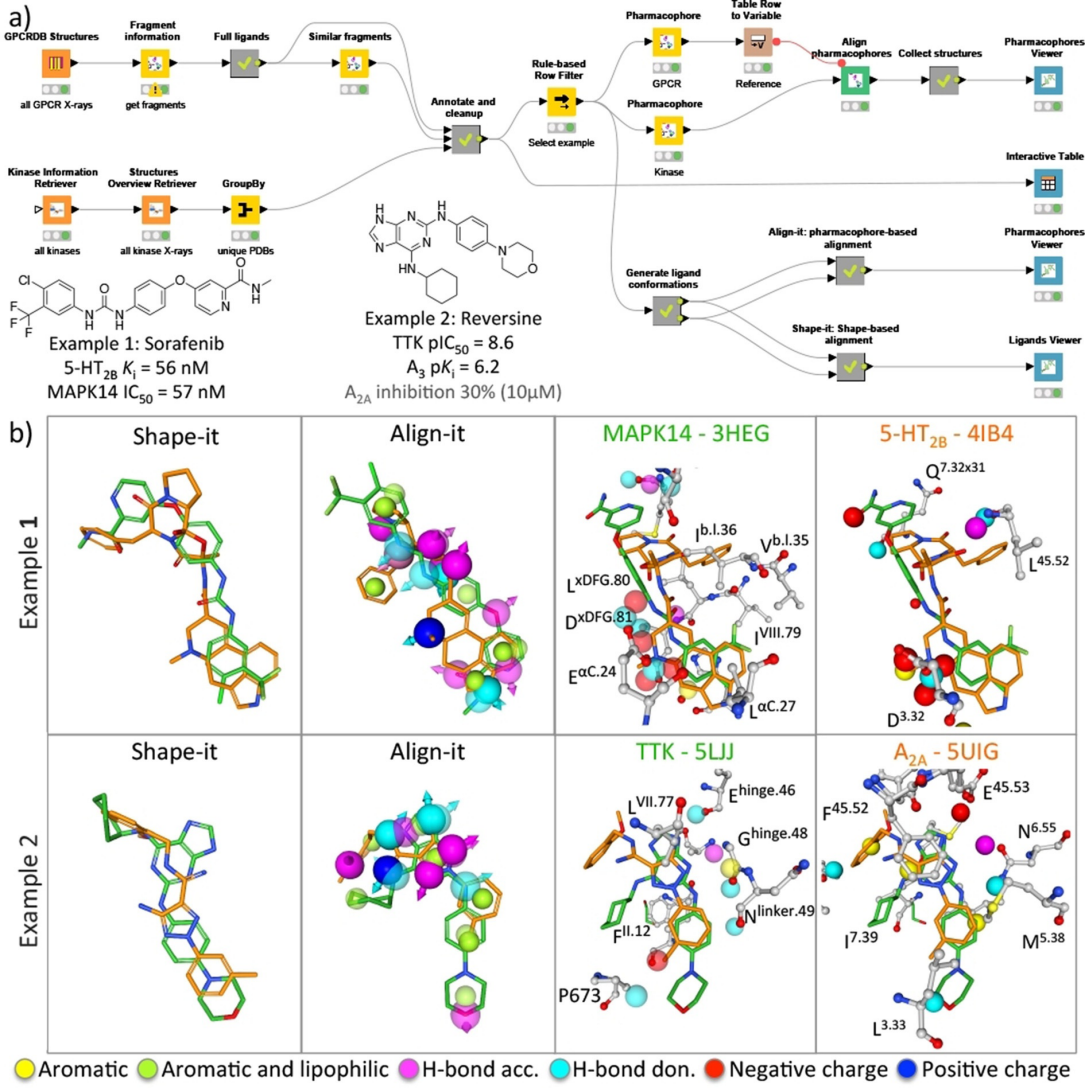
A structure‐based ligand repurposing workflow (A) that searches for KripoDB^26^ pharmacophore similarities between GPCRs and kinases. Two examples (B) of binding site similarities between the 5‐HT_2B_ receptor and MAPK14 kinase, and the adenosine A2A receptor and the TTK kinase are presented and described in the main text. The aligned kinase and GPCR structures based on the alignment of the KRIPO pharmacophores are shown in 3D using the Proteins and Ligands viewer (for clarity purposes the lipophilic pharmacophore features are hidden). Only residues within 3.5 Å of the ligands are depicted and labeled according to the Ballesteros–Weinstein^45^ and KLIFS^24^ numbering scheme for GPCRs and kinases, respectively. Complementary shape‐based and pharmacophore‐based assessment of the ligands using the KNIME‐enabled Silicos‐it^25^ tools Shape‐it and Align‐it are performed and compared in the Ligands viewer and Pharmacophore viewer, respectively.

Ligand repurposing across protein families can be enabled through the comparison of known protein binding sites based on the available crystal structures.^47^ The rationale is that proteins with similar binding sites can potentially bind similar ligands.^47^, ^48^ In this workflow (Figure 6) we compare the KRIPO binding site pharmacophores from all structures of a protein (family) of interest against the KRIPO pharmacophores of the full PDB to identify ligand‐repurposing possibilities.

**Protocol**:

- Collect available PDB entries for the protein families of interest
- Obtain the KRIPO fragments information based on the PDB entries of the reference protein family and search for similar KRIPO fragments in the PDB
- Extract similar fragments that match with PDB entries from the query protein family
- Select interesting fragment pairs and further explore them by, for example, KRIPO pharmacophore alignment and 3D similarity comparison.

With the GPCRdb KNIME nodes, an overview of all GPCR crystal structures^49^ is obtained and used to query the KripoDB26b for the available pharmacophore fragment information for these structures. For all full ligand KripoDB entries a similarity search is performed with the KripoDB similar fragments node. The results are then filtered using the KLIFS nodes with an overview of all kinase crystal structures yielding an overview of GPCR pharmacophore fragments that share similarity with a kinase pharmacophore fragment based on their KripoDB fingerprints. From this list, two examples were selected that identified a possible overlap between the KRIPO pharmacophores based on a kinase and a GPCR structure. The first example is the match between the Sorafenib‐bound MAPK14 protein kinase^50^ (PDB ID: http://www.rcsb.org/pdb/explore/explore.do?structureId=3HEG) and the Ergotamine‐bound 5‐HT_2B_ receptor^51^ (PDB ID: http://www.rcsb.org/pdb/explore/explore.do?structureId=4IB4), consistent with studies showing that the FDA‐approved kinase inhibitor Sorafenib has nanomolar affinity for 5‐HT_2B_R.^52^ The second example is the match between Reversine‐bound TTK protein kinase^53^ (PDB ID: http://www.rcsb.org/pdb/explore/explore.do?structureId=5LJJ) and the triazolecarboximidamide‐bound A_2A_ receptor^54^ (PDB ID: http://www.rcsb.org/pdb/explore/explore.do?structureId=5UIG). Reversine shows weak binding affinity for the adenosine A_2A_ receptor, and has sub‐micromolar affinity for the homologous adenosine A_3_ receptor.^55^

The KRIPO pharmacophores of each structure were downloaded and aligned using the KripoDB pharmacophore and Align Pharmacophores nodes, respectively. The rotational matrix obtained from the alignment was then used to align both pharmacophores as well as the complete PDB entries in the pharmacophore viewer. To compare the structure‐based pharmacophore alignment of the molecules with a ligand‐based approach both molecules were aligned using a ligand‐based pharmacophore approach (Align‐it) and a shape‐based approach (Shape‐it). The SMILES of both co‐crystallized ligands were obtained from the PDB using the PDB Connector Custom Report node. Then the RDkit Add Conformers node was used to generate 30 conformations for each ligand as input for the Align‐it and Shape‐it nodes. The ligand‐based alignments were again visualized with the Pharmacophores Viewer and the Ligands and Proteins viewer. Interestingly, the urea moiety of Sorafenib binding in the back pocket of MAPK14 is aligned with the basic amine in the fused tetracyclic head of Ergotamine. This ligand alignment originates from the KRIPO pharmacophore alignment as the negatively charged centers of the conserved glutamate (E71^αC.24^) in the αC‐helix of MAPK14 and the key aspartate D135^3.32^ of 5‐HT_2B_R are matched.

The volume‐based Shape‐it overlay shows a good overlap (Tanimoto score=0.67) between the two compounds, however, most pharmacophore features are not aligned due to a 180‐degree flip of the core scaffold to maximize the shape overlay. The ligand‐based pharmacophore overlay using Align‐it results in a poor score (Tanimoto score=0.22) and an alignment in which the whole molecules are flipped 180 degrees, illustrating that the structure‐based KRIPO pharmacophores were key for the elucidation of this off‐target effect.

## Conclusions

The presented structural cheminformatics tools and integrated workflows combine heterogeneous data analyses that enable the prediction of protein–ligand interactions and the identification of protein–protein relations. The reusable workflows provide general guidelines that can be used for the construction of automated computer‐aided drug discovery protocols, or for the customization and extension to other targets and applications:

1. The use of well documented and amenable workflow management platforms like KNIME facilitate the construction of consistent, reproducible,^1^ and transferable protocols.7c The workflows can be transferred between, for example, workstations, users, and sites, and can be re‐run: i) as is, for example, when large data transfer is not feasible, or when new database versions are released; ii) with different configurations of the nodes, for example, changing ligand activity cut‐offs (Figure 2), input ligands (Figures 3, 4, 6), protein targets (Figure 5); iii) with additional/modified nodes to obtain complementary information, for example, including annotations from other databases, further analyzing results, or performing machine learning^56^ on the obtained data. Pre‐configured meta nodes or workflow blocks can be easily reused because the same data collection, preparation, processing and analysis steps might be required in various workflows for different purposes.
2. KNIME contains a rich and continuously growing set of cheminformatics nodes to handle and process chemical and biological data in multiple formats. Custom nodes can be developed, such as the nodes presented in the current study, and scripts and external tools can be embedded to extend the functionalities of this toolkit in order to address a plethora of biochemical research questions, for example, structural protein–ligand interaction analysis and prediction functionalities.
3. Carefully annotated and standardized data resources are required to perform integrated cheminformatics analyses.2a, 30, 57 However, it should be noted that the use of external databases can also present a potential pitfall as they can change content and format thereby disrupting the workflow or changing the outcome.
4. The infrastructure of a workflow management platform such as KNIME allows for interactive checks during execution of the workflow. Checking the input and output for each step during the development of a workflow makes for easy debugging resulting a more robust and less error‐prone workflow. To enhance this process customized data visualization nodes, such as the proteins and ligands viewer and the pharmacophore viewer nodes presented in the current study, are also required to inspect the validity of, for example, docking studies, pharmacophore‐based structure alignments, and binding mode similarity assessments.
5. Combining complementary techniques within the same workflow allows for the creation of more advanced or more accurate (consensus)^58^ cheminformatics workflows, for example, by combining ligand‐based on protein–ligand interaction based similarity assessments^59^ or by combining 2D and 3D ligand‐based similarity^60^ methods.

## Experimental Section

**Newly developed KNIME nodes**: The KNIME workflows described in this article use a series of 3D‐e‐Chem KNIME nodes that have been newly developed in addition to a set of previously published 3D‐e‐Chem nodes. An overview of the new nodes is shown in the list below and the nodes themselves are discussed in more detail in the next few paragraphs.

- *Pharmacophore*: Retrieval of the KRIPO pharmacophore based on the KripoDB fragment identifier.
- *Ligands Viewer*: visualization of (aligned) small molecules.
- *Ligands and Proteins Viewer*: the combined visualization of (aligned) small molecules and proteins
- *Proteins Viewer*: visualization of (aligned) proteins
- *Pharmacophores Viewer*: visualization of (aligned) pharmacophores, small molecules and proteins
- *Align pharmacophores*: align the query pharmacophores to the reference pharmacophore.
- *Extract pharmacophore points*: extract the points of a pharmacophore as rows.
- *Merge pharmacophore points*: create pharmacophores from a table with *x*, *y*, *z* coordinates, pharmacophore type, alpha and optional directionality.
- *Pharmacophore from molecule*: create a pharmacophore from a molecule by mapping atoms to pharmacophore points.
- *Pharmacophore to molecule*: generate a molecule from a pharmacophore by mapping pharmacophore points to atoms.
- *Pharmacophore reader*: reads a pharmacophore file (*.phar) in the Silicos‐it phar file format.
- *Pharmacophore writer*: writes a pharmacophore to a file (*.phar) in the Silicos‐it phar file format.
- *PLANTS bindingsite*: calculates the binding site definition for docking based on a reference ligand or pocket atoms of the protein.
- *PLANTS session builder*: takes the protein, binding site and ligands from KNIME and creates the docking session.
- *PLANTS virtual screening*: runs the actual docking itself based on the session created by the session builder.
- *PLANTS virtual screening results reader*: reads the docking results into a KNIME table.
- *Align‐it*: aligns molecules to a reference molecule based on their pharmacophore features and scores the alignment.
- *Align‐it Pharmacophore generator*: generates pharmacophores for molecules based on their pharmacophore features.
- *Filter‐it*: filters a set of molecules based on molecular property ranges.
- *Filter‐it property calculator*: calculates molecular properties for a given set of molecules.
- *Qed Calculator*: performs a quantitative estimation of drug‐likeness (QED) of a set of given molecules. Requires qed.py Python package to be installed
- *Shape‐it*: performs a shape‐based alignment and scoring of a set of ligands to a reference ligand.
- *Strip‐it*: strips a given set of molecules to its scaffold based on a user‐selected scaffold definition.
- *Ss‐TEA score*: calculates the ss‐TEA score for each residue position of a sequence alignment for a set of family members.

Most of the nodes are available under the permissive Apache License 2.0 (https://www.apache.org/licenses/LICENSE-2.0). The PLANTS binaries for docking (embedded within the PLANTS nodes) are freely available for academics, and the Silicos‐it source is available under the GNU Lesser General Public License v3 (https://www.gnu.org/licenses/lgpl‐3.0.en.html). A more detailed overview per node set and tool, including license information, dependencies, and their application, is given in Supporting Information Table S1.

**GPCRdb nodes**: The GPCRdb^23^ is a specialized database focused on G protein‐coupled receptors: the largest protein family that lies encoded within the human genome. Besides a comprehensive ontology, this database contains information on GPCR sequences, alignments, residue numbering schemes, crystal structures, interactions, and mutation data. The eight GPCRdb KNIME nodes, as previously described,^31^ provide access to this information from within KNIME and enable the integration of this data in comprehensive chemogenomics workflows.

**KLIFS nodes**: KLIFS contains kinase‐ligand interaction information derived from over 3900 structures covering more than 270 different kinases in complex with ≈2500 unique ligands (accessed August 2017). All kinase structures within KLIFS are curated, annotated, aligned, and processed in a systematic manner with automated weekly updates. All KLIFS content can be accessed from within KNIME using one or more of the nine KLIFS nodes from four different categories, as published in McGuire et al.^31^

**KripoDB nodes**: The pairwise pharmacophore similarity of more than half a million (sub)pockets extracted from structures in the Protein Data Bank is available in the KripoDB. KRIPO encodes pocket pharmacophores into a fuzzy 3‐point pharmacophore fingerprints that are subsequently used to assess this similarity.26a Besides the “Fragment information” and the “Similar fragments” KRIPO nodes that were previously published,26a a new KripoDB KNIME node has been added for the retrieval of the pharmacophores themselves that where used for the creation of the KRIPO fingerprints. This allows a user to obtain the pharmacophore of interest, and to align and visualize it in combination with the new set of “Pharmacophore” nodes as well as the “Pharmacophores Viewer”.

**Molviewer nodes**: The freely available molecule viewers in KNIME are primarily oriented at visualization of small molecules. To enable displaying proteins, protein–ligand complexes, and pharmacophores in KNIME we created a set of visualization nodes. When opening a KNIME view of one of the new viewer nodes a web browser will be opened with an interactive 3D canvas portraying the input molecule(s). There are four molecule viewer KNIME nodes: one to view a set of (aligned) small molecules (e.g., shape‐it results), one to view a set of (aligned) small molecules and proteins (e.g., for visualizing PLANTS docking results), one to view a set of (aligned) proteins (e.g., obtained from KLIFS), and one to view a set of pharmacophores and their aligned protein and/or ligands (e.g., from aligning KripoDB pharmacophores). The molecule viewer KNIME nodes supports HiLiting, which means that a selection of molecules inside the viewer can be sent to other KNIME nodes and vice versa. The web‐based molecule viewers use the NGL protein viewer^61^ (https://github.com/arose/ngl) as its 3D canvas and use React, Redux, and Bootstrap for controls. The KNIME nodes are written in Java. The web application files are hosted by a Jetty‐based webserver and the Jersey‐based web service, which are both embedded inside the nodes.

**Pharmacophore nodes**: The pharmacophores nodes are a set of KNIME nodes that enable the conversion and alignment of pharmacophores. The nodes support (directed) pharmacophore features with the following supported types: aromatic, H‐bond donor, H‐bond acceptor, lipophilic, positively charged, negatively charged and exclusion. The pharmacophores nodes comprise nodes that read and write pharmacophores in the Silicos‐it phar file format, nodes to convert a pharmacophore from or to a molecule by mapping the pharmacophore types from or to elements, nodes that convert 3D points with a type information into a pharmacophore and vice versa, and finally there is a node to align pharmacophore(s) to a reference pharmacophore. The pharmacophore alignment is performed by comparing all the point pair combinations the pharmacophores can have in common and then identifies the maximum point pair combinations using Bron–Kerbosch^62^ clique detection algorithm. It subsequently uses the Kabsch^63^ algorithm to compute the optimal translation and rotation matrices using singular value decomposition, which are then applied to the probe pharmacophores to get the aligned probe pharmacophores for each point pair combination. The pharmacophore KNIME nodes are written in Java and depend on the ejml Java library (http://ejml.org/) for matrix operations. The alignment algorithm is based on the KRIPO^26^ codebase.

**PLANTS**: PLANTS^27^ is a free‐for‐academics docking tool that employs an ant‐colony optimization algorithm for sampling potential ligand binding modes and uses a semi‐empirical scoring function. The PLANTS KNIME nodes are: i) binding site node to calculate the binding site definition based on the ligand molecule or pocket atoms of the protein, ii) configuration reader to read PLANTS definition files which are used for configuration and to determine the docking output file names, iii) configuration generator to generate a PLANTS config file using the nodes dialog with almost all PLANTS configuration fields divided into tabs, iv) runner, the node that executes the PLANTS executable, v) session builder, which takes the protein, binding site, and ligands from KNIME as input and writes them in a session directory as files as input for the PLANT executable, vi) virtual screening runs the PLANTS executable in screen mode and will read the files written by the session builder, and finally vii) the virtual screening results reader which reads the output files generated by the virtual screening node into KNIME. The PLANTS runner and PLANTS configuration generator KNIME nodes are written in Java and use the Mustache template library^64^ to write the PLANTS config file. All the other PLANTS nodes are implemented as KNIME meta nodes. A PLANTS executable for Windows, Linux and Mac OS X is bundled with the PLANTS KNIME nodes and is provided under a free academic license. The location of the PLANTS executable defaults to the bundled version, but can be overwritten in the KNIME preferences. The initialization and combination of PLANTS KNIME nodes for docking runs requires great care. Therefore, an example docking workflow has been made available at https://github.com/3D-e-Chem/knime-plants/blob/master/examples/plants-virtual-screening-example.knwf.

**Silicos‐it nodes**: Silicos‐it^25^ released several of their cheminformatics tools to the open source domain. These KNIME nodes bring their functionality to the KNIME environment. The nodes are: i) align‐it,^65^ which aligns molecules to a reference molecule based on their pharmacophore, ii) shape‐it,65b,65c, 66 which aligns molecules to a reference molecule based on their shape, iii) filter‐it,^67^ which can filter molecules with undesired properties from a compound set, iv) strip‐it, which generates the Murcko,^68^ Oprea,^69^ or Schuffenhauer^70^ scaffold of a molecule v) Qed,^71^ which calculates the Quantitative Estimation of Drug‐likeness (QED) for a (set of) molecule(s). The Silicos‐it executables are written in C++ and have OpenBabel as a dependency to read and write different molecule formats. The KNIME Silicos‐it nodes come bundled with the align‐it, filter‐it, shape‐it, strip‐it executables for Linux and Mac OS X. The location of the executable defaults to the bundled versions, but can be overwritten in the KNIME preferences. All the Silicos‐it KNIME nodes are implemented as KNIME meta nodes, except for the node that executes the actual Silicos‐it executables. The silicos‐it execute node is implemented in Java and is used by all meta nodes. The align‐it executable is wrapped into two KNIME nodes. A node to align SDF formatted molecules to a reference molecule and another node to generate pharmacophores from molecules. The align‐it KNIME nodes are part of the Silicos‐it KNIME nodes plugin. The shape‐it executable aligns molecules to a reference molecule based on their shape. The shape‐it executable is wrapped in a KNIME node, which aligns SDF formatted molecule to a reference molecule. The output of the node has the aligned molecules and alignment scores.

**ss‐TEA**: The ss‐TEA score^28^ is an abbreviation for subfamily‐specific Two Entropy Analysis score. The score is calculated for each residue position of a large sequence alignment based on a comparison of the level of conservation within a subset (i.e., a subfamily) of proteins (internal entropy) compared to all other proteins (external entropy). By identifying positions that are highly conserved within, but not outside of the subfamily, the ss‐TEA score can identify residue positions specifically related to ligand binding or protein function for that specific subset. This methodology is, however, dependent on a high quality and large quantity sequence alignment as input. The ss‐TEA algorithm has been implemented as a KNIME node, is written completely in Java and has no dependencies. The node requires a sequence alignment and a list of sequence identifiers, which will be used as the subfamily.

**Workflows**: All KNIME workflows described in this article, including the source code for all 3D‐e‐Chem nodes, are available from the 3D‐e‐Chem GitHub repository (https://github.com/3D-e-Chem/workflows). The individual steps of each workflow are described in more detail in the main text. All 3D‐e‐Chem nodes used to perform the analyses described in the current work are available under community contributions in KNIME under “3D‐e‐Chem” (https://www.knime.com/3d-e-chem-nodes-for-knime).

## Conflict of interest

*The authors declare no conflict of interest*.

## Supporting information

As a service to our authors and readers, this journal provides supporting information supplied by the authors. Such materials are peer reviewed and may be re‐organized for online delivery, but are not copy‐edited or typeset. Technical support issues arising from supporting information (other than missing files) should be addressed to the authors.

Supplementary
