# Supplementary material for: 3D‐e‐Chem: Structural Cheminformatics Workflows for Computer‐Aided Drug Discovery
Source: ChemMedChem. 2018 Feb 14;13(6):614–26. doi: 10.1002/cmdc.201700754 (PMC5900740; doi:10.1002/cmdc.201700754)
Supplement: Supplementary file 1 — Supplementary [file CMDC-13-614-s001.pdf]

CHEMMEDCHEM
Chemistry & Drug Discovery

Supporting Information

3D-e-Chem: Structural Cheminformatics Workflows for Computer-Aided Drug Discovery

Albert J. Kooistra+,[a, b] Márton Vass+,[b] Ross McGuire,[a, c] Rob Leurs,[b] Iwan J. P. de Esch,[b] Gert Vriend,[a] Stefan Verhoeven,*[d] and Chris de Graaf*[b]

cmdc_201700754_sm_miscellaneous_information.pdf

Table S1. Structural cheminformatics tools and databases

Method	Type	API [a]	Open source/access	Dependencies[b]	Application [c]
PLANTS[1]	Executable		Free academic license		Ligand binding mode prediction (Fig. 3)
KLIFS[2]	Web application + KNIME nodes	Swagger web service	Apache license 2.0		(Fig. 2, 3, 6)
GPCRdb[3]	Web application + KNIME nodes	Swagger web service	Apache license 2.0	Django, PostgreSQL	(Fig. 4, 5, 6)
PP_GPCR (SEA[4]/OCEAN[5])	Workflow				Ligand repurposing (Fig. 4)
Silicos-it tools	Executables		GNU LGPLv3	OpenBabel 2.0	(Fig. 5)
ss-TEA[6]	KNIME node		Apache license 2.0	-	Binding site identification (Fig. 5)
Molviewer	KNIME nodes	Swagger web service	Apache license 2.0	NGL	Visualize (docked) ligands in a protein (Fig. 4)<br>Visualize (aligned) ligands (Fig. 6)<br>Visualize (aligned) pharmacophores and structures (Fig. 6)
KNIME-pharmacophore	KNIME nodes		Apache license 2.0	EJML	(Fig. 5)
KRIPOdb[7]	Executable + KNIME nodes	Python + Swagger web service	Apache license 2.0	RDKit, PyTables, SQLite	Off-target/polypharmacology prediction (Fig. 5) and identification of bioisosteres.

[a] Application Programming Interface; [b] Software and/or database dependencies; [c] Figures showing the application described in the current work is provided between brackets.

References:

[1] aO. Korb, T. Stützle, T. E. Exner, *Proc. IEEE Swarm Intell. Symp.* **2007**, *1*, 115-134; bO. Korb, T. Stützle, T. E. Exner, *J. Chem. Inf. Model.* **2009**, *49*, 84-96.

[2] aO. P. J. van Linden, A. J. Kooistra, R. Leurs, I. J. P. de Esch, C. de Graaf, *Journal of medicinal chemistry* **2013**, *57*(2), 249-277; bA. J. Kooistra, G. K. Kanev, O. P. van Linden, R. Leurs, I. J. P. de Esch, C. de Graaf, *Nucleic Acids Res* **2016**, *44*(D1), D365-371.

[3] V. Isberg, S. Mordalski, C. Munk, K. Rataj, K. Harpsoe, A. S. Hauser, B. Vroling, A. J. Bojarski, G. Vriend, D. E. Gloriam, *Nucleic Acids Res* **2017**, *45*(5), 2936.

[4] M. J. Keiser, B. L. Roth, B. N. Armbruster, P. Ernsberger, J. J. Irwin, B. K. Shoichet, *Nat Biotechnol* **2007**, *25*(2), 197-206.

[5] P. Czodrowski, W. G. Bolick, *J Chem Inf Model* **2016**, *56*(10), 2013-2023.

[6] M. P. Sanders, W. W. Fleuren, S. Verhoeven, S. van den Beld, W. Alkema, J. de Vlieg, J. P. Klomp, *BMC Bioinformatics* **2011**, *12*, 332.

[7] aT. Ritschel, T. J. Schirris, F. G. Russel, *J Cheminform* **2014**, *6*(Suppl 1), O26; bD. J. Wood, J. de Vlieg, M. Wagener, T. Ritschel, *J Chem Inf Model* **2012**, *52*(8), 2031-2043.
